# Hints to Army Surgeons

**Published:** 1861-10

**Authors:** Charles T. Jackson

**Affiliations:** Boston


### Selections.

### HINTS TO ARMY SURGEONS.

By CHARLES T. JACKSON, M. D., Boston.

*From the Boston Medical and Surgical Journal.*

It may be thought impertinent, in one who is exclusively a chemist, to venture any suggestions for the consideration of surgeons, now employed in the armies of the United States; but it should be remembered that I have spent much of my life in the study of medicine and surgery, and have had uncommonly good opportunities for observing military surgery in France, after the revolution of July, 1830, and in the insurrection of June, 1832. This must be my apology for obtruding a few remarks, which, though they may not be new to many surgeons, will perhaps be useful suggestions to some, and especially to those who have heretofore had no opportunities for improvement in this kind of knowledge.

Gun-shot wounds, I know, have much changed in character since the introduction of improved projectiles—the minié and slug balls in particular; and therefore improved instruments, adapted to the extraction of such balls, and the fragments of bone, should have been prepared. The old-fashioned ball-forceps, adapted to the extraction of a round bullet, are entirely unfit for the extraction of a minié ball or conical slug. Charrier's polypus forceps are vastly preferable; for, instead of dilating at the orifice of a wound, where it is most tender, they actually, for the diameter of any bullet, diminish as to the space they occupy, owing to the crossing of the blades below the fulcrum. This instrument is the best I have ever used for the extraction of a ball, or of fragments of bones, bits of cloth, and other foreign bodies carried into a wound. They are strong enough, and yet are light and portable, serving in place of the common dressing forceps, for all common operations, while they are amply sufficient for the removal of a ball. They are also so light, that they will, in most cases, serve in place of the probe, in explorations, and are long enough to reach to any required depth into a wound. I therefore recommend them to all army surgeons, who, if they are not supplied with them now, will find them to be an exceedingly valuable addition to their operating or dressing cases. I brought from France the first pair of these instruments ever seen in this country, and Dr. John C. Warren borrowed them and had others made here for his own use, and for the Massachusetts General Hospital.

When a round ball strikes a cylindrical bone, it simply breaks it, and not unfrequently the ball itself is split into two pieces, or it is flattened and glides off into the soft parts. The round-ball forceps are not fit for the extraction of the fragments of lead, nor of the comminuted fractured bones. If a round ball strikes a bone, having in its interior a mass of cancellated structure, such, for instance, as the condyles of the knee-joint, the bone is not fractured into fragments, but the ball penetrates the hard shell on the exterior, and beds itself in the cancellated bone, if it does not pass entirely through the joint. If it does pass through, the orifice of exit of the ball will be much larger than where it entered, and there will be some splinters of the hard shell of bone torn off, and the proportion of these fragments will be greater, in direct ratio to the diminished velocity of the ball.

The minié ball, when it strikes the shaft of a long cylindrical bone, generally splits it for a considerable distance, especially if it strikes the bone fairly in the middle. The ball itself is more rarely divided, though it is flattened and compressed sometimes on its sides, where it is not supported, owing to its cavity at the base. If it strikes the side of a bone, it glances to one side very strongly, and will not be found in the direction in which it entered, and it is often very difficult to find where it is lodged, until inflammation reveals the spot.

In order to compare the effects of the common round and the conical minié ball, I took eight one inch pine boards and nailed them together, and then fired the two kinds of balls through the mass of board from a rifled carbine. The round ball made a hole gradually enlarging as it entered, and splintered the board somewhat; but the minié ball entered the first thickness of boards, making a smooth round hole, but split the further half of them all to pieces, tearing off, as it passed out, large splinters of the wood. The effect of a ball, the force of which is partially suspended, will evidently be more destructive then when having its full velocity. It is observed, too, by all who have used a minié rifle, or any of the breech-loading guns, that in close action the men are more likely to overshoot their adversaries, than they would be if they employed the common musket or rifle, not prepared for long range. Indeed, many of the breech-loading guns I have seen, have no provision for sighting at near objects, and it is difficult to hit a mark nearer than one or two hundred yards; therefore soldiers really run much less risk, in charging boldly upon troops employing these weapons, than by carrying on a distant fusilade.

Leaving this subject, let me ask our army surgeons to prepare statistical tables of their operations in which they use anæsthetic agents; whether ether, chloroform, or the mixture of these two anæsthetics. It is very desirable that we should have the means of making a careful comparison of the effects they may produce on the healing of wounds. Ether is known not to delay or prevent healing by first intention. Is it true, also, with regard to chloroform?

We wish, also, by an extensive tabular statement of cases, to compare the mortality of operations under these two anæsthetics, and also with those in which four measures of ether and one measure of chloroform are employed; this being the preparation I have recommended for army use, in case the surgeons cannot carry so bulky an article as ether.

The subjoined table of a few cases, from *Benisson's Méthode Anesthésique*, will perhaps serve as a model for the record, and those who please may add more side columns for such other remarks as they may wish to enter on the record.

TABLE OF CASES.

| No. | Date. | Nature of Operation. | Sex. | Age. | Anæsthetic. | Healed. | Died. |
|---|---|---|---|---|---|---|---|
| | 1847 | | | | | | |
| 1 | March 4 | Amputation left arm.... | man | 43 | Ether. | In 20 ds. | |
| 2 | " 22 | " right leg.... | man | 30 | " | 23 ds. | |
| 3 | April 25 | " left thigh.... | man | 22 | " | 15 ds. | [ritonitis |
| 4 | May 3 | Lithotomy by perineum | boy | 11 | " | | 17 d., pe- |
| 5 | " 28 | Extirp. rt. br'st; cancer. | wom. | 59 | " | | 5th d., |
| 6 | Aug. 12 | " cancer rt breast | wom. | 30 | " | 12 ds. | pleurisy |
| | 1848 | | | | | | |
| 7 | Aug. 3 | Amputation right leg.... | man | 36 | Chloroform. | 15 ds. | |
| 8 | July 10 | " " arm... | man | 23 | " | 20 ds. | |
| 9 | Aug. 3 | Extirp cancer rt breast.. | wom. | 29 | " | 1 mo. | |
| 10 | March 3 | Cancer of the lip, tumor involving the sub-hyoidean region......... | man | 40 | " | | 15 ds. after opera. |

Remarks may be added in full, by reference to the numbers, so that all the particulars which cannot be conveniently tabulated may be introduced ; such as a description of the disease for which the operation was performed, the condition of the patient, the time which the operation required, the treatment after the operation, and, in case of death, the opinion of the surgeon as to the cause.

